# *Tessaria absinthioides* (Hook. & Arn.) DC. (Asteraceae) Decoction Improves the Hypercholesterolemia and Alters the Expression of LXRs in Rat Liver and Hypothalamus

**DOI:** 10.3390/metabo11090579

**Published:** 2021-08-27

**Authors:** Mariana Rey, María S. Kruse, Rocío N. Magrini-Huamán, Jessica Gómez, Mario J. Simirgiotis, Alejandro Tapia, Gabriela E. Feresin, Héctor Coirini

**Affiliations:** 1 Laboratorio de Neurobiología, Instituto de Biología y Medicina Experimental (IBYME-CONICET), Ciudad Autónoma de Buenos Aires (CABA) C1428ADN, Vuelta de Obligado 2490, Argentina; mariana.rey@ibyme.conicet.gov.ar (M.R.); sol.kruse@conicet.gov.ar (M.S.K.); nahimemagrini@gmail.com (R.N.M.-H.); 2 Instituto de Biotecnología-Instituto de Ciencias Básicas, Universidad Nacional de San Juan (UNSJ), Av. Libertador General San Martín 1109 (O), San Juan CP 5400, Argentina; jesicagomez674@gmail.com (J.G.); atapia@unsj.edu.ar (A.T.); gferesin@unsj.edu.ar (G.E.F.); 3 Facultad de Ciencias Médicas, Universidad Católica de Cuyo, Av. José Ignacio de la Roza 1516, San Juan 5400, Argentina; 4 Instituto de Farmacia, Facultad de Ciencias, Campus Isla Teja, Universidad Austral de Chile, Valdivia 5090000, Chile; mario.simirgiotis@gmail.com; 5 Center for Interdisciplinary Studies on the Nervous System (CISNe), Universidad Austral de Chile, Valdivia 5090000, Chile; 6 Consejo Nacional de Investigaciones Científicas y Técnicas (CONICET), CABA, Godoy Cruz 2290 (C1425FQB), Argentina

**Keywords:** brain lipid metabolism, caffeoylquinic acid, dietary fat, nuclear receptors (LXR, PPAR, RXR), UHPLC-PDA-OT-MS/MS phenolic compounds analysis, triglyceride metabolism

## Abstract

Chronic high-fat diet consumption induces hypercholesterolemia. The effect of *Tessaria absinthioides* (Hook. & Arn.) DC. (Asteraceae) was studied on the levels of total cholesterol (TC), high-density lipoprotein cholesterol (HDL-c), and triglycerides, and on the expression of liver X receptors (LXRs) in a hypercholesterolemic model. Adult male rats received a normal diet (ND) or a high-fat diet (HFD; normal diet + bovine fat + cholesterol). After 14 days, rats received water (W) or a decoction of the aerial parts of *T. absinthioides* (Ta; 10% *w*/*v*) for 2, 4, or 6 weeks. Four and six weeks of Ta improved the levels of TC and HDL-c in HFD. After 6 weeks of Ta, the expression of LXRs in HFD was the same as that in ND in both tissues. The Ta chemical profile was studied with an ultrahigh resolution liquid chromatography Orbitrap MS analysis (UHPLC–PDA–OT-MS/MS). Fifty-one compounds were identified, of which twelve are reported for the first time. Among these compounds, caffeoylquinic acid and its derivatives could modify the lipid profile and the expression of LXRs. This is the first *in vivo* report of *T. absinthioides*, which may be a potential candidate against hypercholesterolemia.

## 1. Introduction

The chronic intake of high-fat diets can cause metabolic, cardiovascular, and neurodegenerative diseases [1]. These diets also affect the levels of total cholesterol (TC) and triglycerides (TG), and the expression of liver X receptors (LXRs) in the liver and hypothalamus from rats [2]. LXRs participate in the metabolisms of cholesterol, lipid, fatty acid, and glucose [3]. In addition, LXRs participate in processes related to the steroidogenesis, the immune system, and the inflammatory process [4]. Disturbances on the signaling pathways controlled by LXRs are related to metabolic, neurological, and/or cancerous diseases [5]. There are two LXRs isoforms: LXRα and LXRβ, which are encoded by different genes [3]. The LXRα subtype is expressed mainly in the liver, small intestine, kidney, macrophages, and adipose tissue, whereas the LXRβ subtype is expressed ubiquitously [6]. Both LXRs subtypes are equally able to induce hepatic lipogenesis. However, LXRα is the main subtype in the hepatic pathway [7]. Several reports have revealed that alterations in LXRβ are associated with neurodegenerative diseases [8,9]. Thus, LXRβ is probably the main subtype in the brain.

In the treatment of hypercholesterolemia are used several commercial drugs. However, some of these drugs can cause secondary effects or can be ineffective in some patients [10,11,12]. Several reports have revealed that plants and/or plant-derived compounds (phytosterols, diterpenes, phenolic acids, and flavonoids) reduce the lipid serum and levels of TC, and modulate the expression of LXRs [13,14,15]. Natural products provide molecules that can be pharmacological tools to treat illness [16]. In addition, flavonoids have the ability to differentially modulate the function of LXRs [17]. The plant family Asteraceae has members that have hypocholesterolemic properties even when they are administered during short-term periods [18]. *Tessaria absinthioides* (Hook. & Arn.) DC. (Asteraceae), which is distributed in Argentina, Bolivia, Chile, and Uruguay, is popularly used as infusion or decoction in the treatment of hypercholesterolemia, diabetes and digestive disorders [19,20]. The leaves of *T. absinthioides* have hypocholesterolemic, balsamic, and expectorant properties [21]. In addition, several reports have revealed that *Tessaria sp*. has antimicrobial, antiviral, anti-inflammatory, cytotoxic, antitumoral, and antioxidant properties [22,23,24,25,26,27]. Several works have described that *T. absinthioides* has beneficial compounds, such as 3-β-5-α-dihydroxy costic acid, tessaric acid, thio-phene,2-(but-3-en-1-ynyl)-5-(penta-1-3-diynyl), casticine, artemisine, chrysosplenetine, fatty acids, sesquiterpenes, and phenolic acids [20,21,22,27]. However, the effects of *T. absinthioides* on the homeostasis of cholesterol remain unexplored.

The aim of the present work was to evaluate the effect of a decoction of the aerial parts of the *T. absinthioides* on the levels of TC, high-density lipoprotein cholesterol (HDL-c), and TG, and on the expression of LXRs (in the liver and hypothalamus) in a hypercholesterolemic model. Animals subjected to a high-fat diet [2] received water (W) or a *T. absinthioides* decoction (Ta, 10% *w*/*v*) for 2, 4, or 6 weeks. We hypothesized that some phenolic compounds might improve the lipid profile and modify the expression of LXRs in the animals fed with a high-fat diet. Thus, the full metabolome polyphenolic profile, elucidated with a hybrid high-resolution mass spectrometer (UHPLC-PDA-OT-MS/MS) and the total phenolics and flavonoids content of the Ta were analyzed. This is the first study reporting on the hypocholesterolemic properties of *T. absinthioides in vivo*.

## 2. Results

### 2.1. Body Weight (BW), BW Gain, and the Intake of Beverage and Food

**Figure 1:**
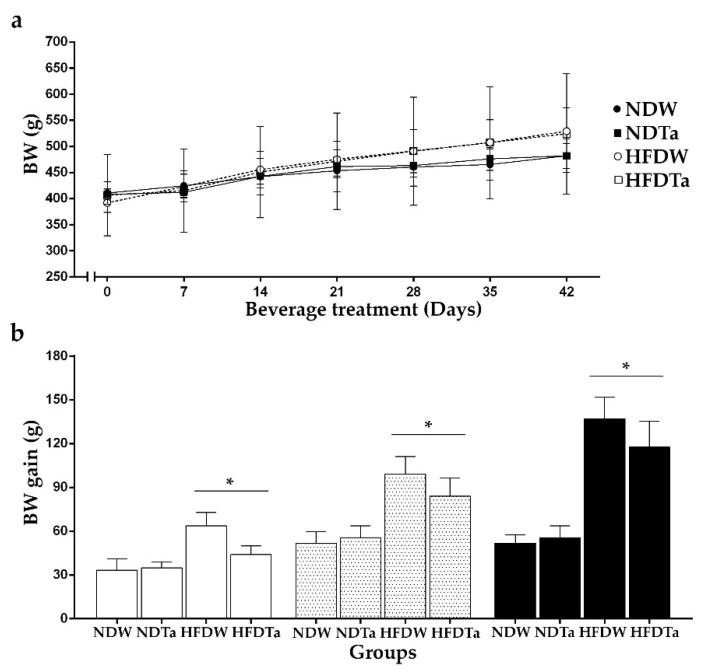
BW (**a**) and BW gain (**b**) after 2 (white bars), 4 (dotted bars), or 6 (black bars) weeks of W or Ta administration. Results are expressed as mean ± SEM from two to three independent assays (*n* = 5 animals/group). Significant differences were determined by two-way ANOVA followed by Newman–Keuls’ post-hoc test. * *p* < 0.05. * refers to the ND in each week period. Abbreviations: NDW, normal diet + water; HFDW, high-fat diet + water; NDTa, normal diet + Ta; and HFDTa, high-fat diet + Ta.

The comparison of the BW of the groups did not show significant differences in each evaluated week (Figure 1a). In contrast, the comparison of the BW gain revealed significant differences. Although the supplementation with Ta did not cause any effect in the evaluated weeks, rats fed with a high-fat diet (HFD) had higher BW gain than those fed with a normal diet (ND; week 2: 57.62%, 4: 70.54%, and 6: 73.94%, *p* < 0.05, Figure 1b, statistics in Table S1, Supplementary Material).

**Figure 2:**
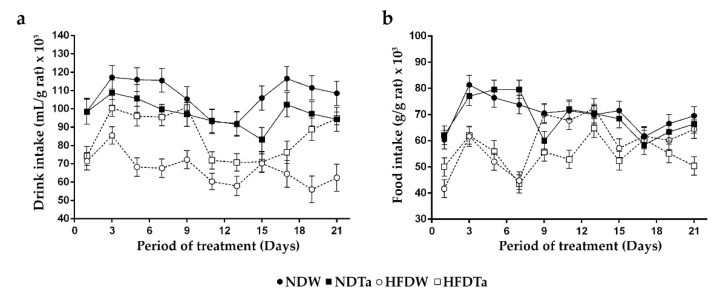
Intake of beverage (**a**) and food (**b**) through a 21-day period since the beverage administration began. Intake values were relativized to the BW of the animals. The graphs include the results of every other day to simplify the figures. Results are expressed as mean ± SEM from two to three independent assays (*n* = 5 animals/group). Significant differences were determined by RM two-way ANOVA followed by Fischer’s LSD post-hoc test. Abbreviations: NDW, normal diet + water; HFDW, high-fat diet + water; NDTa: normal diet + Ta; and HFDTa, high-fat diet + Ta.

The comparison of the beverage intake of the groups revealed significant differences (please see the legend of Figure 2 for abbreviations). HFDW consumed more beverage than NDW in 21 days of the period studied (*p* < 0.05). HFDTa consumed more beverage than NDTa in 3 days of the period studied (*p* < 0.05). In contrast, HFDTa consumed less beverage than NDTa in 11 days of the period studied (*p* < 0.05). In addition, HFDTa consumed the same amount of beverage as NDTa in 7 days of the period studied. NDTa consumed more beverage than NDW in 1 day of the period studied (*p* < 0.05). In contrast, NDTa consumed less beverage than NDW in 16 days of the period studied (*p* < 0.05). In addition, NDTa consumed the same amount of beverage as NDW in 4 days of the period studied. HFDTa consumed more beverage than HFDW in 19 days of the period studied (*p* < 0.05). In addition, HFDTa consumed the same amount of beverage as HFDW in 2 days of the period studied (Figure 2a, statistics in Table S2, Supplementary Material).

The comparison of the food intake of the groups revealed significant differences. HFDW consumed less amount of food than NDW in 13 days of the period studied (*p* < 0.05). In addition, HFDW consumed the same amount of food as NDW in 8 days of the period studied. HFDTa consumed less amount of food than NDTa in 19 days of the period studied (*p* < 0.05). In addition, HFDTa consumed the same amount of food as NDTa in 2 days of the period studied. NDTa consumed more amount of food than NDW in 5 days of the period studied (*p* < 0.05). In contrast, NDTa consumed less amount of food than NDW in 1 day of the period studied (*p* < 0.05). In addition, NDTa consumed the same amount of food as NDW in 15 days of the period studied. HFDTa consumed more amount of food than HFDW in 2 days of the period studied (*p* < 0.05). In contrast, HFDTa consumed less amount of food than HFDW in 10 days of the period studied (*p* < 0.05). In addition, HFDTa consumed the same amount of food as HFDW in 9 days of the period studied (Figure 2b; statistics in Table S2, Supplementary Material).

### 2.2. Levels of TC, HDL-c, and TG

The comparison of the levels of TC, HDL-c, and TG revealed significant differences (*p* < 0.05).

After 2 weeks, the supplementation with Ta did not modify these serum parameters. HFD had higher levels of TC (55.31%) and TG (40.67%) and lower levels of HDL-c (43.39%) than ND (*p* < 0.05, Table 1).

After 4 weeks, HFDW had higher levels of TC (43.36%) and lower levels of HDL-c (31.00%) than NDW (*p* < 0.05). HFDTa had lower levels of TC (24.89%) and higher levels of HDL-c (64.49%) than HFDW (*p* < 0.05). In addition, HFDTa had the same levels of TC and HDL-c as NDTa. The supplementation with Ta did not modify the levels of TG. Thus, HFD had higher levels of TG (51.04%) than ND (*p* < 0.05; Table 1). 

After 6 weeks, HFDW had higher levels of TC (43.29%) and lower levels of HDL-c (33.33%) than NDW (*p* < 0.05). HFDTa had lower levels of TC (34.18%) and higher levels of HDL-c (30.12%) than HFDW (*p* < 0.05). In addition, HFDTa had the same levels of TC and HDL-c as NDTa. The supplementation with Ta did not modify the levels of TG. Thus, HFD had higher levels of TG (69.13%) than ND (*p* < 0.05; Table 1).

NDTa had the same levels of TC, HDL-c, and TG as NDW in all the evaluated weeks (Table 1; statistics in Table S3, Supplementary Material).

### 2.3. The LXRs Expression

**Figure 3:**
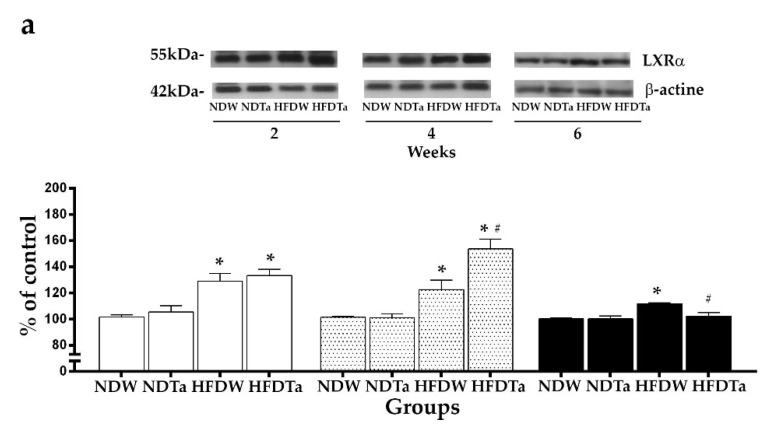

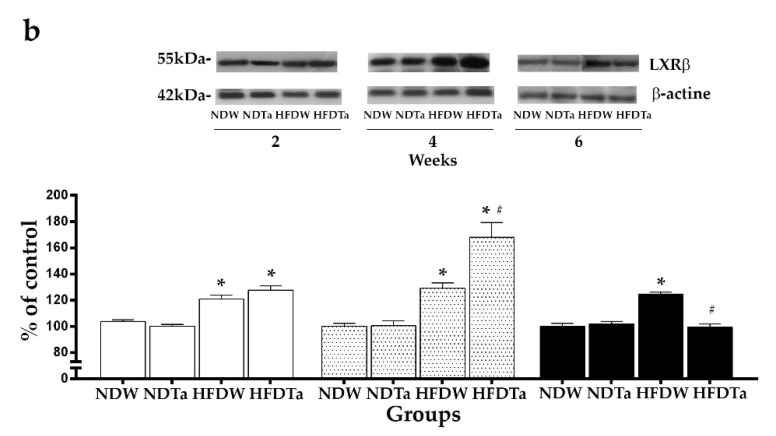
The expression of LXRα (**a**) and LXRβ (**b**) in the liver after the administration of W or Ta for 2 (white bars), 4 (dotted bars), or 6 (black bars) weeks. The expression of LXRs was quantified by Western Blot and represented as a percentage of ND. Representative western blots are showed on the top of each graph. Results are expressed as mean ± SEM from two independent assays (*n* = 5 animals/group). Significant differences were determined by two-way ANOVA followed by Newman–Keuls’ post-hoc test. * refers to each respective ND and ^#^ refers to the HFDW. *^,#^ *p* < 0.05 Newman–Keuls’ post-hoc test. Abbreviations: NDW, normal diet + water; HFDW, high-fat diet + water; NDTa, normal diet + Ta; and HFDTa, high-fat diet + Ta.

The comparison of the expression of LXRα and LXRβ revealed significant differences in the liver and hypothalamus. After 2 weeks, the supplementation with Ta did not modify the hepatic expression of the LXRs. HFD had higher levels of expression of LXRs than ND (LXRα: 26.80% and LXRβ: 21.97%, *p* < 0.05; Figure 3). After 4 weeks, HFDW had higher levels of expression of LXRs than NDW (LXRα: 20.83% and LXRβ: 29.01%, *p* < 0.05, Figure 3). HFDTa had higher levels of expression of LXRs than NDTa (LXRα: 52.03% and LXRβ: 67.17%, *p* < 0.05). In addition, HFDTa had higher levels of expression of LXRs than HFDW (LXRα: 25.39% and LXRβ: 30.02%, *p* < 0.05, Figure 3). After 6 weeks, HFDW had higher levels of expression of LXRs than NDW (LXRα: 11.55% and LXRβ: 24.67%, *p* < 0.05, Figure 3). HFDTa had the same levels of expression of LXRs as NDTa. In addition, HFDTa had lower levels of expression of LXRs than HFDW (LXRα: 8.45% and LXRβ: 20.36%; *p* < 0.05; Figure 3).

**Figure 4:**
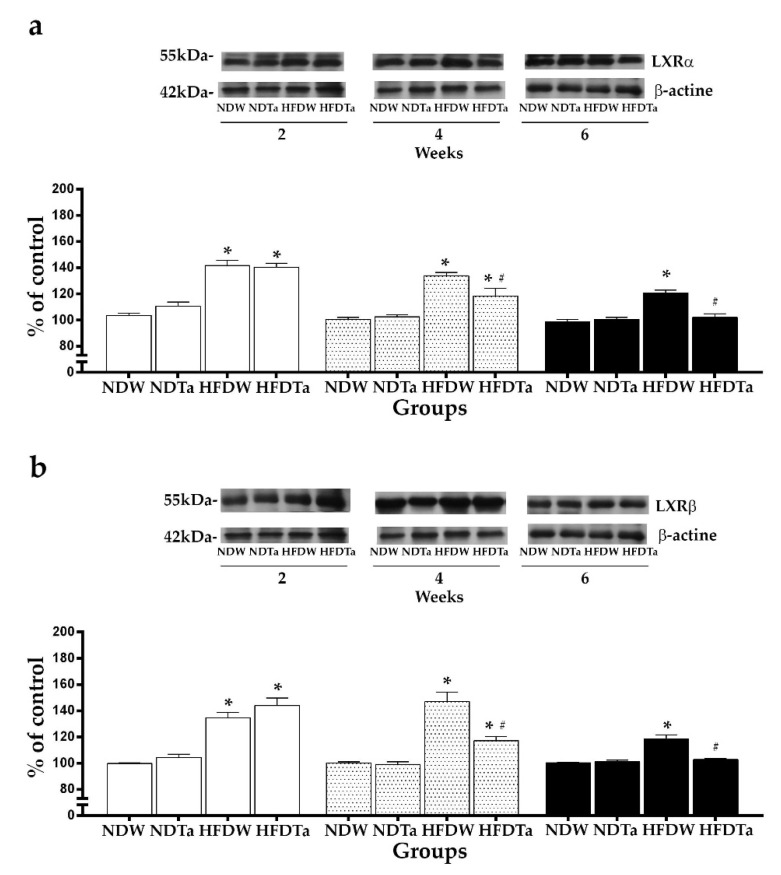
The expression of LXRα (**a**) and LXRβ (**b**) in the hypothalamus after the administration of W or Ta for 2 (white bars), 4 (dotted bars), or 6 (black bars) weeks. The expression of LXRs was quantified by Western Blot and represented as a percentage of ND. Representative western blots are showed on the top of each graph. Results are expressed as mean ± SEM from two independent assays (*n* = 5 animals/group). Significant differences were determined by two-way ANOVA followed by Newman–Keuls’ post-hoc test. * refers to each respective ND and ^#^ refers to the HFDW. *^,#^ *p* < 0.05 Newman–Keuls’ post-hoc test. Abbreviations: NDW, normal diet + water; HFDW, high-fat diet + water; NDTa: normal diet + Ta; and HFDTa, high-fat diet + Ta.

Regarding to the hypothalamus, after 2 weeks, the supplementation with Ta did not modify the expression of the LXRs. HFD had higher levels of expression of LXRs than ND (LXRα: 31.90% and LXRβ: 36.48%, *p* < 0.05; Figure 4). After 4 weeks, HFDW had higher levels of expression of LXRs than NDW (LXRα: 32.92% and LXRβ: 46.80%, *p* < 0.05, Figure 4). HFDTa had higher levels of expression of LXRs than NDTa (LXRα: 15.21% and LXRβ: 18.13%, *p* < 0.05). However, HFDTa had lower levels of expression of LXRs than HFDW (LXRα:11.60% and LXRβ: 19.84%, *p* < 0.05, Figure 4). After 6 weeks, HFDW had higher levels of expression of LXRs than NDW (LXRα: 22.18% and LXRβ: 18.90%, *p* < 0.05, Figure 4). HFDTa had the same levels of expression of LXRs as NDTa. In contrast, HFDTa had lower levels of expression of LXRs than HFDW (LXRα: 15.30% and LXRβ: 13.41%; *p* < 0.05; Figure 4).

NDTa had the same levels of expression of LXRs as NDW in all the tissues and evaluated weeks (Figure 3 and Figure 4; statistics in Table S4, Supplementary Material).

### 2.4. UHPLC-OT Analysis of Ta 

**Figure 5:**
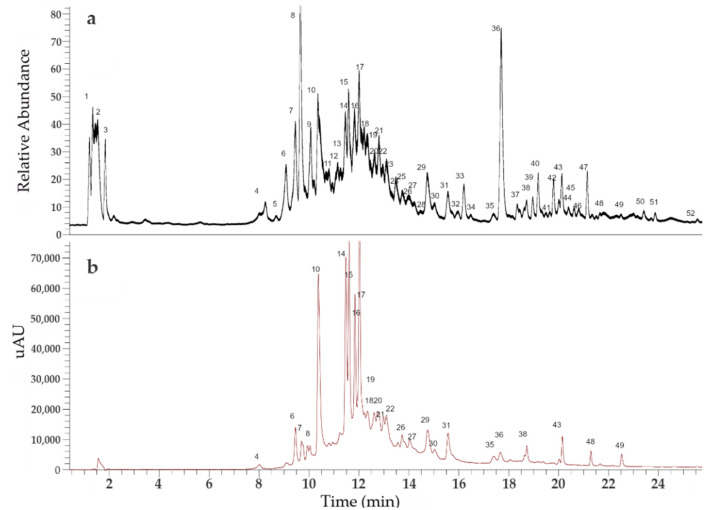
UHPLC chromatograms of Ta: (**a**) The total Ion Current (TIC) chromatogram and (**b**) the UV-vis chromatogram at 280 nm.

The ultrahigh resolution liquid chromatography Orbitrap MS analysis UHPLC–PDA–OT-MS/MS combining full MS spectra and MSN experiments revealed the presence of fifty-one compounds in Ta, of which twelve of them are reported for first time (27, 29, 31, 33, 37, 41, 43, 44, 45, 49, 50, and 51). The composition of Ta included phenolics acids, fatty acids, and several characteristic eudesmane sesquiterpenoids. Some of these compounds were identified by spiking experiments with available standards. Compounds with a phenolic -OH easily lose the proton in electrospray ionization, providing very good diagnostic parent ions and fragments. The analyses were confirmed using MS/MS data and by comparing the fragments found with the available bibliography of this species [20,22,27]. Databases such as SciFinder, MassBank of North America (MoNA), Spectrabase (Wiley), and UHPLC-MS internal library (tessaric acid, Ilicic acid, 3,4-Dihydroxy-costic acid, 10-Undecenoic acid, 3-oxo-gamma costic acid, gamma costic acid, trihy-droxy-octadecadienoic acid, and 2,3,4-Trihydroxyoctadeca-2,4-dienoic acid) were used. Figure 5 showed a UHPLC-MS (total ion current) and UV chromatograms of Ta.

The analysis revealed the presence of fifty-one compounds in the metabolome of Ta (Table 2). Among these compounds (Figure 5) were eight phenolic acids such as caffeoylquinic acid (CQA; peak 6), vanillic acid (peak 8), and di-CQA isomers (1′,5′; 3′,5′ and 4′,5′ di-CQA; peaks 14, 15, and 17). In addition, the anti-inflammatory compounds 3′,4′5′ tri-CQA (peak 20), ginnalin A (peak 26), and related tetra-CQA (peak 30) were found in the profile.

Thirteen typical compounds were closely assigned to sesquiterpenes. Several of them had the eudesmane skeleton and were previously reported as constituents of this plant, such as the derivative 5,3,4,7 tetrahydroxypentosyl tessaric acid (peak 9); hymenoxynin (peak 11); ilicic acid (peak 16); tessaric acid (peak 35) and its isomers (peaks 38 and 40); plusthe eudesmane 4(15) and 11(13)-dien-12, 5βb-olide (peak 19); and the matricarin sesquiterpene phenolic compound scorzonerin (peak 32). Meawhile, peak 39 was identified as alpha costic acid (peak 50), alongside its isomer gamma costic acid (peak 48) and derivative 3-oxo-gamma costic acid (peak 42); two isomers of 3,4 and 3,5-dihydroxy-costic acid (Peaks 24 and 28); and 5-acetyl, 3-hydroxy-4- reduced dihydro-costic acid (peak 39).

Six compounds were identified as polyhydroxylated unsaturated fatty acids such as trihydroxy-octadecadienoic acids (peak 33 and 34); trihydroxy-octadecaenoic acid (peak 36) and the saturated diacid 3-hydroxyoctanedioic acid (3-hydroxysuberic acid; peak 7); peak 51 as 2-hydroxydocosanoic acid; and peak 29 as 10-undecenoic acid. Other compounds tentatively identified were quinic acid (peak 1); citric acid (peak 3); manoheptulose (peak 2); bruecceantin (peak 18); peak 27 as caffeoyl feruloylquinic acid; peak 31 as sambucinol; peak 43 as chysosplenetin; peak 44 as 3-Acetyl,5-hydroxy-4 dihydrocosticacid; peak 45 as geranyl propionate; peak 46 as eupatorine; and peaks 4, 5, 10, 12, 13, 21, 23, 25, 47, and 52 remain unknown.

Ta presented a high total phenolic (TP) content with a value of gallic acid equivalents (1.96 ± 0.01 mg GAE/mL Ta), which approximately 9% of them corresponds to flavonoids (0.18 ± 0.01 mg quercetin equivalents (QE)/mL Ta). 

## 3. Discussion

The chronic consumption of high-fat diets can cause dyslipidemia and metabolic, cardiovascular and neurodegenerative diseases [1]. Several drugs are used in the treatment of hyperlipidemia. However, some of these drugs can be ineffective or can cause adverse reactions [11]. Thus, the search of new treatments is a major concern. In this work, we evaluated the actions of a decoction of *T. absinthioides* in rats fed with a high-fat diet [2].

At the evaluated weeks, the supplementation with Ta did not modify the BW of the groups. In addition, the values of BW were similar to those obtained in hypercholesterolemic animals after a supplementation with “Silymarin” (a mixture of flavonolignans extracted from *Silybum marianum* (L.) Gaertn (Asteraceae)) and a supplementation with *Taraxacum officinale* G. H. Weber ex Wigg. (Asteraceae) [18,28].

The supplementation with Ta did not cause any effect on BW gain (Figure 1). However, the co-administration of Ta with a high-fat diet caused in BW gain a non-significant tendency to decrease similarly to that reported with the administration of *Stevia rebaudiana* Bertoni (Asteraceae) [29].

The supplementation with Ta modified the intake of beverage and food. Ta increased the beverage intake and decreased the food intake in the animals fed with the high-fat diet. In contrast, Ta decreased the beverage intake and did not modify the food intake in the animals fed with the normal diet (Figure 2). In addition, the animals fed with the high-fat diet consumed less beverage and food than the animals fed with the normal diet. In the bibliography of family members of Asteraceae are reported different observations on the same hypercholesterolemic model. Krečman et al. (1998) have revealed that a supplementation with “Silymarin” does not change the intake of food [18], whereas Ahmad et al. (2018) have revealed that a supplementation with *Stevia rebaudiana* decreases the intake of beverage and food [29]. 

We have previously described that 14 days of a high-fat diet modify the lipid profile [2]. In this work, 2 weeks of a supplementation with Ta did not modify the variations in the levels of TC, HDL-c, and TG found in animals fed with a high-fat diet. However, other reports have found that several family members of Asteraceae improve the hypercholesterolemia state even in short-term administrations. Al-Jubouri et al. (1990) have described that 10 days of a supplementation with *Chamomillarecutita* (L.) Rauschert (Asteraceae) reduce the levels of TC but not the levels of TG in hyperlipidemic rats [30]. Krečman et al. (1998) have described that a supplementation with “Silymarin” reduces the levels of TC and increases the levels of HDL-c in animals fed with a high-fat diet [18]. 

Four and six weeks of the supplementation with Ta reduced the levels of TC and increased the levels of HDL-c in the animals fed with the high-fat diet (Table 1). However, the supplementation with Ta did not modify the levels of TG independently of the evaluated week. Some of these results are in agreement with the available bibliography. Choi et al. (2010) have found that 4 weeks of supplementation with *Taraxacum officinale* do not modify the levels of TC, but increase the levels of HDL-c and reduce the levels of TG in hypercholesterolemic animals [28]. Ahmad et al. (2018) have revealed that 8 weeks of supplementation with *Stevia rebaudiana* reduce the levels of TC and TG, and increase the levels of HDL-c in rats fed with a high-fat diet [29].

The hypocholesterolemic properties of *T. absinthioides* decoction observed in the present work could be attributable to the combined effect of flavonoids, fatty acids, sesquiterpenes, and phenolic acids, which have reported hypolipidemic properties [13,20,22,27]. The flavonoids promote an increase in fecal sterols that in turn leads to decreased absorption of dietary cholesterol [31,32]. In addition, the consumption of flavonoids increases the levels of HDL-c that removes the cholesterol from peripheral tissues to the liver for catabolism and excretion [17,33]. In addition, flavonoids and polyphenols increase the cholesterol metabolism and modulate the enzymes involved in this process [34]. The dietary supplementation with plant sterols may reduce the levels of TC. Compounds with a chemical structure similar to cholesterol, such as sitosterol, stigmasterol, campesterol, brasicasterol, and ergosterol, are poorly absorbed in the intestine. In addition, plant sterols inhibit the absorption of cholesterol, displacing it from bile micelles [32,35].

We have previously described that the consumption of a high-fat diet modifies the expression of LXRs in the liver and hypothalamus [2]. In this work, 2 weeks of supplementation with Ta did not modify the expression of LXRs in the animals fed with the high-fat diet. In contrast, 4 weeks of supplementation with Ta increased the hepatic expression of LXRs and reduced the hypothalamic expression of LXRs in the animals fed with the high-fat diet. Six weeks of supplementation with Ta returned the expression of LXRs to normal levels in the animals fed with the high-fat diet (Figure 3 and Figure 4). These results reinforce our previous findings that suggest that the hypothalamic LXRs have a role in lipid homeostasis [2,36]. In addition, these results seem to suggest that supplementation with Ta can modulate the expression of LXRs and the lipid metabolism associated to these receptors. The effects on the expression of LXRs may be attributed to the presence of diterpenes, phenolic acids, and stanols (phytosterols/phytostanols) in *T. absinthioides*, which can modulate the activity of LXRs [13,16,37,38]. Some terpenes, such as diterpenes, can modulate the activation and repression functions of both LXRs and the cholesterol efflux similarly to synthetic agonists in macrophages [38]. The ultrahigh resolution liquid chromatography Orbitrap MS analysis UHPLC–PDA–OT-MS/MS of Ta revealed the presence of fifty-one compounds, of which twelve (27, 29, 31, 33, 37, 41, 43, 44, 45, 49, 50, and 51) are reported for first time. Among these compounds, the caffeoylquinic acid (CQA; peak 6); di-CQA isomers (1′,5′; 3′,5′ and 4′,5′ di-CQA; peaks 14, 15, and 17); 3′,4′,5′ tri-CQA (peak 20); and tetra-CQA (peak 30) were identified by means of UHPLCMS/MS. Huang (2014) has reported that caffeoylquinic acid can modulate the hepatic expression of LXRα and improve the lipid metabolism disorders observed in a high-fat diet model [13].

In summary, the hypercholesterolemia constitutes a health risk concern because it promotes deleterious effects on the peripheral and central systems. Thus, the search of new therapeutic treatments is an important issue that remains in force. In this work, *T. absinthioides* improves the hypercholesterolemia in animals fed with a high-fat diet. The phenolic compounds present in *T. absinthioides*, identified by means of UHPLC MS/MS, could explain the results observed in these animals. However, the precise mechanism of how *T. absinthioides* causes these effects requires molecular and mechanistic studies. The present work adds relevant information concerning how a plant derivative can help to handle hypercholesterolemia accompanied by peripheral and central changes in the expression of LXRs. In addition, the exhaustive UHPLCMS/MS study updates the chemical profile of this South American medicinal species. We consider that this is the first work that studies *T. absinthioides* in an animal hypercholesterolemic model involving the use of a preparation form similar to that used by people in the traditional medicine. Further studies are required to determine if *T. absinthioides* can be proposed as a new source of beneficial phytocompounds and to extrapolate the hypocholesterolemic effects of *T. absinthioides* in humans. 

## 4. Materials and Methods 

### 4.1. Chemicals

Ultra-pure water (<5 µg/L TOC) was obtained from a water purification system Arium 126 61316-RO plus an Arium 611 UV unit (Sartorius, Goettingen, Germany). Methanol (HPLC grade) and formic acid (puriss. p.a. for mass spectrometry) were obtained from J. T. Baker (Phillipsburg, NJ, USA). Chloroform (HPLC grade) was obtained from Merck (Santiago, Chile). HPLC standards (citric acid, vanillic acid, and chlorogenic acid, all standards with purity higher than 95% by HPLC) were obtained from Sigma-Aldrich Chem. Co. (St Louis, MO, USA) or Extrasynthèse (Genay, France).

### 4.2. Preparation of the Decoction of Tessaria absinthioides

The naturally grown *T. absinthioides* was collected in the locality “Médano de Oro”, Rawson district, San Juan, Argentina, during the conditioning process of a farm. The aerial parts of *T. absinthioides* were dried at room temperature and stored in the absence of light and heat. A voucher specimen was deposited in the “Laboratorio de Productos Naturales of the Universidad Nacional de San Juan” (voucher number IBT-TA-2). The Ta was prepared at 10% *w*/*v* with dried and milled aerial parts of the plant, and the water was purified with PSA equipment. After 30 min of boiling, the Ta was filtered and the volume lost by evaporation was recovered with purified water. The obtained decoction of *T. absinthioides* (Ta) was stored at −20 °C for further use.

### 4.3. Ultrahigh Resolution Liquid Chromatography Orbitrap MS Analysis UHPLC–PDA–OT-MS/MS of Ta 

#### 4.3.1. UHPLC-DAD-MS Instrument

Liquid chromatography was performed using an UHPLC C18 column (Acclaim, 150 mm × 4.6 mm ID, 2.5 µm, Thermo Fisher Scientific, Bremen, Germany) operated at 25 °C. The detection wavelengths were 280, 254, 330, and 354 nm, and photodiode array detectors were set from 200 to 800 nm. Mobile phases were 1% formic aqueous solution (A) and acetonitrile 1% formic acid (B). The gradient program started at 5% B at zero time; maintained 5% B for 5 min; went to 30% B for 10 min; maintained 30% B for 15 min; went to 70% B for 5 min; maintained 70% B for 10 min; and finally returned to initial conditions in 10 and 12 min for column equilibration before each injection. The flow rate was 1.00 mL min^−1^ and the injection volume was 10 µL. Standards were dissolved in methanol and the decoction were kept at 10 °C during storage in the autosampler. The HESI II and Orbitrap spectrometer parameters were optimized as previously reported [17]. Briefly: sheath gas flow rate, 75 units; auxiliary gas unit flow rate, 20; capillary temperature, 400 °C; auxiliary gas heater temperature, 500 °C; spray voltage, 2500 V (for ESI-); and S lens, RF level 30. Full scan data in positive and negative were acquired at a resolving power of 70,000 FWHM at *m*/*z* 200; scan range of *m*/*z* 100–1000; automatic gain control (AGC) was set at 3 × 106; and the injection time was set to 200 ms. The chromatographic system was coupled to MS with a source II heated electro-nebulization ionization probe (HESI II). The nitrogen gas carrier (purity > 99.999%) was obtained from a Genius NM32LA (Peak Scientific, Billerica, MA, USA) generator and used as a collision and damping gas. The mass calibration for Orbitrap was performed every day in order to ensure the accuracy of an operating mass equal to 5 ppm. Mass calibration for Orbitrap was performed in both negative and positive modes once a day to ensure working mass 5 ppm of accuracy. For the positive mode, a mixture of caffeine (1 mg/mL, 20 µL) and N-butylamine (1 mg/mL, 100 µL) was used, whereas for the negative mode, a mixture of sodium dodecyl sulfate (1 mg/mL, 100 µL) and taurocholic acid sodium salt (1 mg/mL, 100 µL; Sigma-Aldrich, Darmstadt, Germany) was used. In addition, Ultramark 1621 (Alpha Aezar, Stevensville, MI, USA) was used as the reference compound (1 mg/mL, 100 µL). These compounds were dissolved in a mixture of acetic acid (100 µL), acetonitrile (5 mL), water: methanol (1:1; 5 mL; Merck, Santiago, Chile), and 20 µL of the mixture infused using a Chemyx Fusion (Thermo Fisher Scientific, Bremen, Germany) 100 µL syringe pump, and mass calibration performed every day. The Q Exactive 2.0 SP 2, Xcalibur 2.3, and Trace Finder 3.2 (Thermo Fisher Scientific, Bremen, Germany) were used for UHPLC mass spectrometer control and data processing. 

#### 4.3.2. Determination of Total Phenolics and Flavonoids Content 

The total phenolics and flavonoids content in Ta was determined by means of the Folin–Ciocalteu and AlCl_3_ tests, respectively [22]. The results were obtained using calibration curves with standards of the gallic acid (GA) and quercetin (Q), and were expressed as the equivalent in milligrams of these per volume of Ta (mg GA/mL Ta for phenolics compounds and mg Q/ mL Ta for flavonoids). The values, obtained from triplicates, were reported as the mean ± SD. The determinations were made in triplicates using a Multiskan FC Microplate Photometer (Thermo Scientific, Waltham, MA, USA).

### 4.4. Animals, Diets, and Experimental Procedure

Adult male Sprague-Dawley rats (60-day-old, 300–400 g; *n* = 65–70) were housed under standard laboratory conditions in a temperature and humidity-controlled vivarium with a 12-h light–dark cycle and *ad libitum* access to food and water. All procedures concerning animal care and use were carried out according to the European Community Council Directive (86/609/EEC) and the guidelines of the National Institutes of Health Guide for the Care and Use of Laboratory Animals, and were approved by the ethical committee of IBYME (CE060, 9 January 2015) in CABA, Argentina.

The animals were subjected to a normal diet (3.3 kcal/g, protein (18.2%), carbohydrates (56.9%), lipids (3.9%), vitamins and minerals (3%), fiber (5%), and humidity (13%); Gepsa feeds, Grupo Pilar SA, Pilar, Argentina) from 21-day-old to 60-day-old. Then, the animals were equally divided into two groups. One group was subjected to the same normal diet (ND), whereas the second group was subjected to an experimental high-fat diet (HFD) to induce a hypercholesterolemia [2]. The high-fat diet was prepared as we previously described (4.8 kcal/g; normal diet (60%) + bovine refined fat (38%; Productos Reciento, Dinamarg SA, Buenos Aires, Argentina) + cholesterol (2%; sc-202539S, Santa Cruz Biotechnology, Dallas, TX, USA; Table S5, Supplementary Material). After 14 days of the high-fat diet or normal diet, the supplementation with beverages (W or Ta) was administered for 2, 4, or 6 weeks. Thus, the animals were divided into four groups NDW, HFDW, NDTa, and HFDTa (*n* = 5 each one). The beverages (W or Ta) were replaced over 2 days. The non-toxic effects of Ta have been previously reported on healthy individuals [27]. 

The body weight of the animals (BW) was recorded weekly between 09:00–10:00 a.m. from the beginning of the supplementation with W or Ta to the end of the longest supplementation (6 weeks). The BW gain was determined as the difference between the BW at 2, 4, or 6 weeks and the BW before the supplementation with W or Ta (day 14 of the high-fat diet). The intake of beverage and food was measured daily at 10:00 a.m. in the groups of the longest supplementation (6 weeks). The intake of beverage and food was measured in a representative period of 21 days since the beginning of the supplementation with W or Ta. 

The intake of beverage (mL) and food (g/kcal) was relativized to BW. 

The animals were sacrificed after 2, 4, or 6 weeks of the supplementation with W or Ta. On the day of the sacrifice, the animals were fasted for 8 h prior to take blood samples from the tail vein and then the animals were rendered unconscious by CO_2_ and killed by decapitation. The liver and hypothalamus were dissected, frozen, and stored at −80 °C [2,39,40]. 

The results of BW, BW gain, and the intake of beverage and food in the HFDW and HFDTa were compared to those of their respective control (NDW or NDTa).

### 4.5. Lipid Profile

The levels of TC, HDL-c, and TG were determined in blood samples as we previously described [2]. The levels of TC, HDL-c, and TG in the HFDW and HFDTa were compared to the NDW or NDTa, respectively.

### 4.6. Determination of Protein Expression

The expression of LXRs was quantified by Western Blot in homogenates of the liver or hypothalamus as we previously described [2,40]. The primary antibodies were anti LXRα (rabbit, 1:1000) and anti LXRβ (goat, 1:1000). The primary antibody anti β-Actin (goat, 1:3000) was used as the protein loading control. The expression of the proteins was referred to as a percentage of the control group (NDW) in each tissue. All the antibodies were obtained from Santa Cruz Biotechnology, USA. The expression of LXRs in the HFDW and HFDTa was compared to the NDW or NDTa, respectively.

### 4.7. Statistical Analysis

The data were statistically analyzed using the commercial software GraphPad Prism (GraphPad Software Inc., v.4, San Diego, CA, USA), Statview (SAS Institute Inc. v5.0.1, Cary, NC, USA), or SPSS (IBM SPSS statistics, v.21, Armonk, NY, USA). The significant differences between BW, BW gain, the levels of TC, HDL-c and TG, and the expression of LXRs were determined by two-way ANOVA with the factors diet (normal diet and high-fat diet), beverage (W and Ta), and duration (2, 4, and 6 weeks), followed by Newman-Keuls’ post-hoc test. The significant differences between diets were determined by one-way ANOVA. The significant differences in the intake of beverage and food were determined by two-way RM ANOVA with the factor diet (normal diet and high-fat diet) and beverage (W and Ta) with the IBM SPSS statistics software, followed by Fisher’s LSD post-hoc test. RM ANOVA assumptions were tested with Box’s test of equality of covariance matrices and Mauchly’s test of sphericity. In all the cases, the sphericity was significant (*p* < 0.05); thus, the Greenhouse–Geisser correction was reported. The statistical data are presented in the Supplementary Material. For all the cases, the data were expressed as mean ± SEM and differences were considered significant at *p* < 0.05.

## 5. Conclusions

A current health concern regards hypercholesterolemia because it causes deleterious effects on the peripheral and central systems. The search of new therapeutic treatments is an important issue that remains in force. Ta improves the hypercholesterolemia in animals fed with a high-fat diet. This is the first time that *T. absinthioides* is studied in an animal model involving a preparation similar to that used in traditional medicine. The total phenolic and flavonoids content and other compounds present in Ta, identified by means of UHPLCMS/MS, could explain the observed results. However, the molecular and mechanistic explanation of the beneficial effects of *T. absinthioides* requires further studies. The present work adds relevant information concerning how a plant derivative can help to handle hypercholesterolemia accompanied by peripheral and central changes in the expression of LXRs. In addition, the UHPLCMS/MS study updates the chemical profile of this South American medicinal species. Additional studies are required to propose *T. absinthioides* as a new source of beneficial phytocompounds and to extrapolate these effects to humans.

## Figures and Tables

**Table 1:** Changes in lipid parameters after the administration of Ta.

Lipid Parameter	Group	Weeks		
		2	4	6
TC (mg/dL)	NDW	48.80 ± 2.90	48.20 ± 1.70	49.20 ± 0.60
	NDTa	47.60 ± 5.30	46.10 ± 2.30	47.60 ± 1.90
	HFDW	74.70 ± 5.50 *	69.10 ± 6.30 *	70.50 ± 5.00 *
	HFDTa	75.00 ± 5.60 *	51.90 ± 3.40 ^#^	46.40 ± 13.2 ^#^
HDL-c (mg/dL)	NDW	22.30 ± 2.90	20.00 ± 3.10	24.90 ± 3.20
	NDTa	23.10 ± 3.40	22.60 ± 2.70	23.40 ± 2.90
	HFDW	13.10 ± 2.30 *	13.80 ± 2.50 *	16.60 ± 2.90 *
	HFDTa	12.60 ± 2.50 *	22.70 ± 2.90 ^#^	21.60 ± 3.20 ^#^
TG (mg/dL)	NDW	141.60 ± 2.19	141.00 ± 2.91	147.50 ± 3.10
	NDTa	126.90 ± 1.76	161.40 ± 3.78	139.60 ± 3.10
	HFDW	184.50 ± 2.13 *	223.90 ± 9.20 *	248.80 ± 19.8 *
	HFDTa	193.20 ± 1.04 *	238.30 ± 5.70 *	238.30 ± 2.13 *

Levels of TC, HDL-c, and TG in animals fed with the normal diet or high-fat diet after the administration of W or Ta for 2, 4, or 6 weeks. Results are expressed as mean ± SEM from two to three independent assays (*n* = 5 animals/group/week). Significant differences were determined by two-way ANOVA in each individual week followed by Newman–Keuls’ post-hoc test. * refers to the ND in each week and ^#^ refers to the HFDW in each week. *^,#^ *p* < 0.05 Newman–Keuls’ post-hoc test. Abbreviations: NDW, normal diet + water; HFDW, high-fat diet + water; NDTa, normal diet + Ta; and HFDTa, high-fat diet + Ta.

**Table 2:** High resolution UHPLC PDA-Q orbitrap identification of metabolites from Ta.

Peak Number	Retention Time (min)	UV Max	Tentative Identification	Elemental Composition [M-H]	Measured Mass (*m*/*z*)	Theoretical Mass (*m*/*z*)	Ac Curacy (δppm)	MS^n^ Ions (δppm)
1	1.21	-	Quinicacid *	C_7_H_11_O_6_^−^	191.05579	191.05501	4.03	144.00844
2	1.31	-	Manoheptulose *	C_7_H_13_O_7_^−^	209.06633	209.06558	3.37	153.01857
3	1.82	-	Citric acid *	C_6_H_7_O_7_^−^	191.01863	191.01939	3.76	144.00844
4	7.26	-	Unknown	C_16_H_11_O_15_N_3_^−^	365.01859	365.01847	0.23	
5	8.47	330	Unknown	C_4_H_7_O_12_^−^	246.99167	246.99320	−6.2	152.01080
6	8.73	239–320	Caffeoylquinicacid (chlorogenicacid) *	C_16_H_17_O_9_^−^	353.08671	353.08786	3.86	275.0235, 191.05481 (quinicacid), 707.18115 (2M-H)
7	9.55	223	3-Hydroxysuberic acid	C_8_H_13_O_5_^−^	189.07645	189.07575	3.69	
8	10.03	330	Vanillic acid *	C_9_H_7_O_4_^−^	179.03465	179.03389	4.28	135.04436
9	10.07	283	5,3,4,7 tetrahydroxypentosyltessaric acid	C_20_H_31_O_10_^−^	431.19241	431.19117	2.87	311.11367, 135.04433
10	10.36	330	Unknown	C_10_H_9_O_15_N_3_^−^	411.00293	411.00282	0.32	
11	10.43	288–346	Hymenoxynin	C_21_H_34_O_9_^−^	429.21313	429.21191	2.85	267.21184 (M-hexosemoiety)
12	11.12	330	Unknown	C_15_H_7_O_11_N^−^	377.00092	377.00136	−1.18	
13	11.48	330	Unknown	C_10_H_9_O_15_N_3_^−^	411.00296	411.00282	0.34	
14	11.62	239–320	1′,5′ Di-caffeoyl quinic acid (cynarin)	C_25_H_23_O_12_^−^	515.11945	515.11840	2.02	191.05551 (quinic acid), 179.03429
15	11.82	239–320	3′,5′ Di-caffeoyl quinic acid *	C_25_H_23_O_12_^−^	515.11951	515.11840	2.14	191.05562 (quinic acid), 179.03429
16	11.90	255–354	Ilicic acid *	C_15_H_23_O_3_^−^	251.16516	251.16417	4.5	233.15470 (M-H_2_O), 207.17544 (M-CO_2_),171.95076
17	12.00	239–320	4′,5′ Di-caffeoyl quinic acid	C_25_H_23_O_12_^−^	515.11840	515.11945	2.14	191.05551 (quinic acid), 179.03429
18	12.20	335	Bruceantin	C_28_H_36_O_11_^−^	547.21739	547.21832	1.70	
19	12.21	255–365	Eudesmane 4(15), 11(13)-dien-12, 5βolide *	C_15_H_19_O_3_^−^	247.13380	247.13287	3.77	205.15968 (M-CO_2_),149.09645
20	12.57	289–329	3′,4′,5′ Tri-caffeoylquinic acid	C_34_H_39_O_15_^−^	677.15033	677.15119	14.30	515.11963 (Di-CQA), 191.05561(quinic acid)
21	12.81	278	Unknown	C_30_H_33_O_15_N_10_^−^	773.13715	773.21214	−0.08	
22	12.96	278	Unknown	C_26_H_8_ON_10_^−^	476.08734	476.08771	−0.76	
23	13.21	278	Unknown	C_12_HO_16_N_14_^−^	953.17645	953.17632	−0.98	476.08722
24	13.46	278	3,4-Dihydroxy-costic acid *	C_15_H_21_O_4_^−^	265.14459	265.14344	4.32	247.13395 (M-H_2_O)
25	14.04	278	Unknown	C_28_H_23_O_12_N_9_^−^	677.15039	677.14741	4.40	
26	14.70	323	Ginnalin A	C_20_H_19_O_13_^−^	467.08200	467.08202	−0.03	249.08006
27	14.29	213, 287, 326	1-O-Caffeoyl-5-O-feruloylquinic acid	C_26_H_25_O_12_^−^	529.13405	529.13519	2.15	191.05577 (quinicacid), 134.03664
28	16.20	278	3,5-Dihydroxy-costic acid	C_15_H_21_O_4_^−^	265.14456	265.14344	4.24	247.13392(M-H_2_O)
29	16.72	225	10-Undecenoic acid *	C_43_H_35_O_18_^−^	227.12779	227.11839	2.64	183.13847
30	15.55	265–329	Tetra caffeoylquinic acid	C_43_H_35_O_18_^−^	839.17999	839.18289	3.43	191.05552 (quinicacid), 179.03423
31	15.93	335	Sambucinol	C_15_H_21_O_4_^−^	265.14344	265.14453	4.13	
32	15.94	235	Scorzonerin	C_30_H_36_O_11_^−^	571.21872	571.21739	1.63	467.08185, 327.21765
33	16.45	335	2,3,4-Trihydroxyoctadeca-2,4-dienoic acid	C_18_H_31_O_5_^−^	327.21660	327.21783	3.77	183.13832
34	16.48	222	Trihydroxy-octadecadienoic acid	C_18_H_31_O_5_^−^	327.21790	327.21660	3.95	283.22787(M-CO_2_)
35	17.69	283	Tessaric acid *	C_15_H_19_O_3_^−^	247.13379	247.13287	3.71	205.15979 (M-CO_2_), 149.09644
36	18.34	222	Trihydroxy-octadecaenoic acid	C_18_H_33_O_5_^−^	329.23225	329.23364	4.22	285.24352(M-CO_2_)
37	18.69	335	Jaceidin	C_18_H_15_O_8_^−^	359.07614	359.07745	3.64	
38	19.09	283	Tessaric acid isomer	C_15_H_19_O_3_^−^	247.13383	247.13287	3.90	205.15972 (M-CO_2_),163.11223
39	19.25	218	5-Acetyl, 3-hydroxy-4 dihydrocostic acid	C_17_H_25_O_5_^−^	309.17090	309.16965	4.03	291.16019(M-H_2_O), 267.16018(M-acetylmoiety)152.08374
40	19.47	283	Tessaric acid isomer	C_15_H_19_O_3_^−^	247.13383	247.13287	3.90	205.15979 (M-CO_2_),162.01357
41	19.80	320	Artelasticin	C_30_H_33_O_6_^−^	489.24282	489.24405	2.5	245.99947
42	19.96	283	3-oxo-gamma costic acid *	C_15_H_19_O_3_^−^	247.13383	247.13287	3.90	231.13903, 233.11812, 219.13902, 215.00955, 149.09644
43	20.14	335	Chrysosplenetin	C_19_H_17_O_8_^−^	373.09313	373.09317	3.68	310.40393
44	20.39	335	3-Acetyl,5-hydroxy-4 dihydro costic acid	C_17_H_25_O_5_^−^	309.16965	309.17099	4.33	
45	21.12	335	Geranylpropionate	C_15_H_21_O_2_^−^	209.15361	209.15439	3.73	
46	21.53	218	Eupatorin	C_18_H_15_O_8_^−^	343.08258	343.08123	−4.11	329.06663 (M-CH_3_),315.0533 (M-2CH_3_), 313.03531
47	22.53	218	Unknown	C_13_H_27_O_8_^−^	311.16876	311.17004	−4.11	
48	23.41	335	Gamma costic acid *	C_15_H_21_O_2_^−^	233.15453	233.15451	3.87	215.00955, (M-H_2_O)205.15973(M-CO)
49	23.41	335	Gamma costic acid isomer	C_15_H_21_O_2_^−^	233.15453	233.15361	3.94	215.00955, (M-H_2_O)205.15973(M-CO)
50	23.97	335	Alpha Costic acid	C_15_H_21_O_2_^−^	233.15451	233.15361	3.87	215.00953,(M-H_2_O)205.15965 (M-CO)
51	24.61	320	2-Hydroxydocosanoic acid	C_22_H_43_O_3_^−^	355.32067	355.32175	3.02	

* identified by spiking experiments with authentic standards.

## Data Availability

Data is contained within the article or Supplementary Material.

## Supplementary Materials

The following are available online at https://www.mdpi.com/article/10.3390/metabo11090579/s1, Table S1: Two-way ANOVA statistical data of each individual week for BW and BW gain using the factors diet (normal diet or high-fat diet) and beverage (W or Ta), Table S2: RM two-way ANOVA statistical data for food and beverage intake in a 21 day-period using the factors diet (normal diet or high-fat diet) and beverage (W or Ta), Table S3: Two way ANOVA statistical data of each individual week for TC, HDL-c and TG using diet (normal diet or high-fat diet) and beverage (W or Ta) as factors, Table S4: Two-way ANOVA statistical data of each individual week for the expression of LXRs in liver and hypothalamus using the factors diet (normal diet or high-fat diet) and beverage (W or Ta), Table S5: Composition of normal diet and high-fat diet.
