# Supplementary material for: Tessaria absinthioides (Hook. & Arn.) DC. (Asteraceae) Decoction Improves the Hypercholesterolemia and Alters the Expression of LXRs in Rat Liver and Hypothalamus
Source: Metabolites. 2021 Aug 27;11(9):579. doi: 10.3390/metabo11090579 (PMC8467473; doi:10.3390/metabo11090579)
Supplement: Supplementary file 1 [file metabolites-11-00579-s001.zip › metabolites-1299588-supplementary.pdf]

Rey et al., Supplementary material Metabolites 2021

Tessaria absinthioides (Hook. &Arn.) DC. (Asteraceae) Decoction Improves the Hypercholesterolemia and Alters the Expression of LXRs in Rat Liver and Hypothalamus

Mariana Rey, María S. Kruse, RocíoN. Magrini-Huamán, Jessica Gómez, Mario J. Simirgiotis, Alejandro Tapia, Gabriela E. Feresin and Héctor Coirini.

Table S1: Two-way ANOVA statistical data of each individual week for BW and BW gain using the factors diet (normal diet or high-fat diet) and beverage (W or Ta).

	**Weeks**		
	**2**	**4**	**6**
**BW**	$F_{Diet}(1,16)=0.230$; $p=0.6384$<br>$F_{Beverage}(1,16)=0.008$; $p=0.9285$<br>$F_{Interaction}(1,16)=0.015$; $p=0.9056$	$F_{Diet}(1,16)=1.180$; $p=0.2934$<br>$F_{Beverage}(1,16)=0.002$; $p=0.9667$<br>$F_{Interaction}(1,16)=0.003$; $p=0.9549$	$F_{Diet}(1,16)=2.367$; $p=0.1435$<br>$F_{Beverage}(1,16)=0.007$; $p=0.9353$<br>$F_{Interaction}(1,16)=0.007$; $p=0.9353$
**BW gain**	$F_{Diet}(1,16)=7.897$; $p=0.0126$,<br>$F_{Beverage}(1,16)=1.662$; $p=0.2157$<br>$F_{Interaction}(1,16)=2.285$; $p=0.1502$	$F_{Diet}(1,16)=13.458$; $p=0.0021$<br>$F_{Beverage}(1,16)=0.303$; $p=0.5894$<br>$F_{Interaction}(1,16)=0.848$; $p=0.3708$	$F_{Diet}(1,16)=18.724$; $p=0.0005$<br>$F_{Beverage}(1,16)=0.546$; $p=0.4708$<br>$F_{Interaction}(1,16)=0.685$; $p=0.4202$

Table S2: RM two-way ANOVA statistical data for food and beverage intake in a 21 day-period using the factors diet (normal diet or high-fat diet) and beverage (W or Ta).

**Beverageintake**	$F_{Days}(2.97,47.44)=363.610$; $p<0.0001$
	$F_{Days\text{-}Diet}(2.97,47.44)=153.654$; $p<0.0001$
	$F_{Days\text{-}Beverage}(2.97,47.44)=102.687$; $p<0.0001$
	$F_{Days\text{-}Beverage\text{-}Diet}(2.97,47.44)=119.647$; $p<0.0001$
**Foodintake**	$F_{Days}(1.27,20.21)=286.282$; $p<0.0001$
	$F_{Days\text{-}Diet}(1.27,20.21)=275.496$; $p<0.0001$
	$F_{Days\text{-}Beverage}(1.27,20.21)=90.844$; $p<0.0001$
	$F_{Days\text{-}Beverage\text{-}Diet}(1.27,20.21)=67.722$; $p<0.0001$

Table S3: Two way ANOVA statistical data of each individual week for TC, HDL-c and TG using diet (normal diet or high-fat diet) and beverage (W or Ta) as factors.

	Weeks		
	2	4	6
**TC**	$F_{Diet}(1,16)=145.370$; $p<0.0001$<br>$F_{Beverage}(1,16)=0.043$; $p=0.8382$<br>$F_{Interaction}(1,16)=0.118$; $p=0.7359$	$F_{Diet}(1,16)=60.923$; $p<0.0001$<br>$F_{Beverage}(1,16)=31.695$; $p<0.0001$<br>$F_{Interaction}(1,16)= 19.605$; $p=0.0004$	$F_{Diet}(1,16)=9.938$; $p=0.0062$<br>$F_{Beverage}(1,16)=16.259$; $p=0.0010$<br>$F_{Interaction}(1,16)=12.558$; $p=0.0027$
**HDLc**	$F_{Diet}(1,16)=62.085$; $p<0.0001$<br>$F_{Beverage}(1,16)=0.008$; $p=0.9311$<br>$F_{Interaction}(1,16)=0.269$; $p=0.6109$	$F_{Diet}(1,16)=5.862$; $p=0.0277$<br>$F_{Beverage}(1,16)=20.708$; $p=0.0003$<br>$F_{Interaction}(1,16)=6.172$; $p=0.0244$	$F_{Diet}(1,16)=13.977$; $p=0.0018$<br>$F_{Beverage}(1,16)=1.717$; $p=0.2086$<br>$F_{Interaction}(1,16)=5.860$; $p=0.0277$
**TG**	$F_{Diet}(1,16)=44.322$; $p<0.0001$<br>$F_{Beverage}(1,16)=0.138$; $p=0.7150$<br>$F_{Interaction}(1,16)=2.036$; $p=0.1728$	$F_{Diet}(1,16)=53.235$; $p<0.0001$<br>$F_{Beverage}(1,16)=2.528$; $p=0.1314$<br>$F_{Interaction}(1,16)=0.075$; $p=0.78883$	$F_{Diet}(1,16)=99.716$; $p<0.0001$<br>$F_{Beverage}(1,16)=0.839$; $p=0.3734$<br>$F_{Interaction}(1,16)=0.016$; $p=0.9007$

Table S4: Two-way ANOVA statistical data of each individual week for the expression of LXRs in liver and hypothalamus using the factors diet (normal diet or high-fat diet) and beverage (W or Ta).

	Weeks		
	**2**	**4**	**6**
**LIVER**			
**LXRα**	$F_{Diet}(1,16)=45.106$; $p<0.0001$	$F_{Diet}(1,16)=45.037$; $p<0.0001$	$F_{Diet}(1,16)=13.607$; $p=0.0020$
	$F_{Beverage}(1,16)=0.907$; $p=0.3550$	$F_{Beverage}(1,16)=7.845$; $p=0.0128$	$F_{Beverage}(1,16)=6.506$; $p=0.0214$
	$F_{Interaction}(1,16)= 0.002$; $p=0.9590$	$F_{Interaction}(1,16)=8.206$; $p=0.0112$	$F_{Interaction}(1,16)=6.236$; $p=0.0238$
**LXRβ**	$F_{Diet}(1,16)=77.693$; $p<0.0001$	$F_{Diet}(1,16)=55.812$; $p<0.0001$	$F_{Diet}(1,16)=27.977$; $p<0.0001$
	$F_{Beverage}(1,16)=0.381$; $p=0.5456$	$F_{Beverage}(1,16)=9.163$; $p=0.0080$	$F_{Beverage}(1,16)=31.692$; $p<0.0001$
	$F_{Interaction}(1,16)=4.221$; $p=0.0566$	$F_{Interaction}(1,16)=8.847$; $p=0.0089$	$F_{Interaction}(1,16)=42.444$; $p<0.0001$
**HYPOTHALAMUS**			
**LXRα**	$F_{Diet}(1,16)=125.590$; $p<0.0001$	$F_{Diet}(1,16)=50.174$; $p<0.0001$	$F_{Diet}(1,16)=31.181$; $p<0.0001$
	$F_{Beverage}(1,16)=0.861$; $p=0.3672$	$F_{Beverage}(1,16)=3.857$; $p=0.0672$	$F_{Beverage}(1,16)=15.962$; $p=0.0010$
	$F_{Interaction}(1,16)=1.815$; $p=0.196$	$F_{Interaction}(1,16)=6.478$; $p=0.0216$	$F_{Interaction}(1,16)=23.530$; $p=0.0002$
**LXRβ**	$F_{Diet}(1,16)=104.798$; $p<0.0001$	$F_{Diet}(1,16)=61.718$; $p<0.0001$	$F_{Diet}(1,16)=40.049$; $p<0.0001$
	$F_{Beverage}(1,16)=3.784$; $p=0.0695$	$F_{Beverage}(1,16)=13.911$; $p=0.0018$	$F_{Beverage}(1,16)=22.519$; $p=0.0002$
	$F_{Interaction}(1,16)=0.425$; $p=0.5238$	$F_{Interaction}(1,16)=12.236$; $p=0.0030$	$F_{Interaction}(1,16)=28.156$; $p<0.0001$

Table S5:Composition of normal diet and high-fat diet.

	Normal diet	High-fat diet
Proteins	18.2%	15.3%
Carbohydrates	56.9%	39.2%
Lipids	3.9%	1.9%
BovineFat	0	27.5%
Fiber	5%	3.5%
Cholesterol	0	1.4%
Mineral &Vitaminmix	3%	2.5%
Humidity	13%	8.7%
Energy kcal/kg diet	3285	4824

Values are expressed as percent of diet.

Minerals concentration (per kg)	Normal diet	High-fat diet
Manganese (Mn)	75 ppm	53.6 ppm
Iron (Fe)	50 ppm	35.7 ppm
Copper (Cu)	7.4 ppm	5.3 ppm
Iodine (I)	0.6 ppm	0.4 ppm
Calcium (Ca)	1.2 %	0.9 %
Phosphorus (P)	0.9 %	0.6 %
Salt (NaCl)	0.45 %	0.3 %
Sodium (Na)	0.2 %	0.1 %
Zinc (Zn)	55 ppm	39.3 ppm
Seleniun (Se)	0.21 ppm	0.2 ppm

Vitamins concentration (per kg)	Normal diet	High-fat diet
A	6.05 UI	4.3 UI
D3	5.06 UI	3.6 UI
E	22 UI	15.7 UI
C	125 mg	89.3 mg
Niacin	32.34 mg	23.10 mg
Pantothenicacid	19.8 mg	14.1 mg
Thiamine	11 mg	7.9 mg
Riboflavin	3.74 mg	2.67 mg
Menadione	3.08 mg	2.20 mg
Folicacid	2.4 mg	1.7 mg
Pyridoxine	1.9 mg	1.4 mg
Biotin	0.15 mg	0.11 mg
Cyanocobalamin	0.005 mg	0.004 mg

Amino acids concentration (per kg)	Normal diet	High-fat diet

Arginine	1.18 %	0.84 %
Isoleucine	0.75 %	0.54 %
Leucine	1.65 %	1.18 %
Lysine	1.03 %	0.74 %
Methionine - Cystine	0.80 %	0.57 %
Cystine	0.45 %	0.32 %
Methionine	0.34 %	0.24 %
Tryptophan	0.20 %	0.14 %
Threonine	0.69 %	0.49 %

List of the components used in the elaboration of normal diet:Fish flour, meat flour, viscera flour, beef tallow, soybean flour, sunflower flour, corn, wheat, sorghym, barley, oats, roasted bean soybean, bran wheat, gluten flour, sodium chloride, shell, bentonite, calcium phosphate, ash bone, minerals (sodium selenite, calcium iodate, copper oxide, zinc oxide, manganese oxide, ferrous sulfate), vitamins (A, D3, E, C, menadione, thiamine, riboflavin, pyridoxine, pantothenic acid, biotin, niacin, folic acid, cyanocobalamin), liquid choline, amino acids (arginine, isoleucine, leucine, L-lysine, methionine-cystine, cysteine, DL-methionine, tryptophan, threonine), antioxidant (ethoxyquin).
